# Synthesis, structure and Hirshfeld surface analysis of a coordination compound of cadmium acetate with 2-amino­benzoxazole

**DOI:** 10.1107/S2056989023007399

**Published:** 2023-08-30

**Authors:** Surayyo Razzoqova, Aziz Ibragimov, Batirbay Torambetov, Shakhnoza Kadirova, Tamas Holczbauer, Jamshid Ashurov, Bakhtiyar Ibragimov

**Affiliations:** a National University of Uzbekistan named after Mirzo Ulugbek, 4 University St, Tashkent 100174, Uzbekistan; b Institute of General and Inorganic Chemistry, Academy of Sciences of Uzbekistan, M. Ulugbek Str 77a, Tashkent 100170, Uzbekistan; c Institute of Organic Chemistry, Research Centre for Natural Sciences, 2 Magyar tudosok korutja, H-1117 Budapest, Hungary; d Institute of Bioorganic Chemistry, Academy of Sciences of Uzbekistan, M. Ulugbek Str 83, Tashkent 100125, Uzbekistan; Texas A & M University, USA

**Keywords:** crystal structure, mol­ecular structure, cadmium complex, 2-amino­benzoxazole, Hirshfeld surface analysis

## Abstract

A first coordination compound of 2-amino­benzoxazole (**2AB**), namely, bis­(2-amino­benzoxazole-κ*N* ^3^)bis­(acetato-κ^2^ *O*,*O*′)cadmium(II), [Cd(CH_3_COO)_2_(**2AB**)_2_], has been synthesized from ethanol solutions of Cd(CH_3_(COO)_2_ and **2AB**. In the monoclinic crystals with the space group *C*2_1_/*c*, the cadmium ions coordinate two neutral **2AB** mol­ecules in a monodentate fashion through the oxazole N atom, while two acetate ligands are coordinated through the O atoms in a bidentate manner. The coordination polyhedron of the central ion is substanti­ally distorted octa­hedral. There are two relatively strong intra­molecular hydrogen bonds in the complex mol­ecule. Additionally, two inter­molecular hydrogen bonds associate complex mol­ecules into columns running in the [1  0] and [110] directions. The Hirshfeld surface analysis shows that 45.7% of the inter­molecular inter­actions are from H⋯H contacts, 24.7% are from O⋯H/H⋯O contacts and 18.8% are from C⋯H/H⋯C contacts, while other contributions are from N⋯H/H⋯N and O⋯O contacts.

## Abstract 2

The mol­ecular and crystal structures of a cadmium acetate coordination compound with 2-amino­benzoxazole were studied, and the Hirshfeld surfaces and fingerprint plots were generated to investigate various inter­molecular inter­actions.

## 1. Chemical context

Benzoxazole is an aromatic organic compound with a ben­zene-fused oxazole ring structure and an odour similar to pyridine (Katritzky *et al.*, 2000 ▸; Clayden *et al.*, 2001 ▸). Although benzoxazole itself is of little practical inter­est, many benzoxazole derivatives are commercially important. They play an important role in medicinal and biological chemistry (Potashman *et al.*, 2007 ▸; Lachtova *et al.*, 2018 ▸; Razzoqova *et al.*, 2022 ▸), being described as potential therapeutic agents, including as various enzyme inhibitors (Chikhale *et al.*, 2018 ▸). Amino­benzoxazoles, in particular derivatives of 2-amino­benzoxazole (**2AB**), have anti­cancer and anti­bacterial properties (Khajondetchairit *et al.*, 2017 ▸; Ouyang *et al.*, 2012 ▸). The 2-amino-5-chloro­benzoxazole derivative is a muscle relaxant and it has been used as an anti­spasmodic and uricosurics drug (Lynch, 2004 ▸).

An analysis of the Cambridge Structural Database (CSD, Version 5.43, update of March 2022; Groom *et al.*, 2016 ▸) showed that there are no X-ray structures of **2AB** and its metal complexes in the database. However, recently, we reported the structure and inter­molecular inter­actions of a **2AB**–fumaric acid organic salt in which the N atom of **2AB** is protonated by a fumaric acid H atom (Razzoqova *et al.*, 2022 ▸). Theoretically, metal complexes of **2AB** may involve coordination through the N or O atoms of the oxazole ring and the N atom of the amino substituent. In order to define which way these possibilities will be realized, we have prepared a coordination complex of **2AB** with cadmium and report here its mol­ecular and crystal structure, as well as a Hirshfeld surface analysis. 
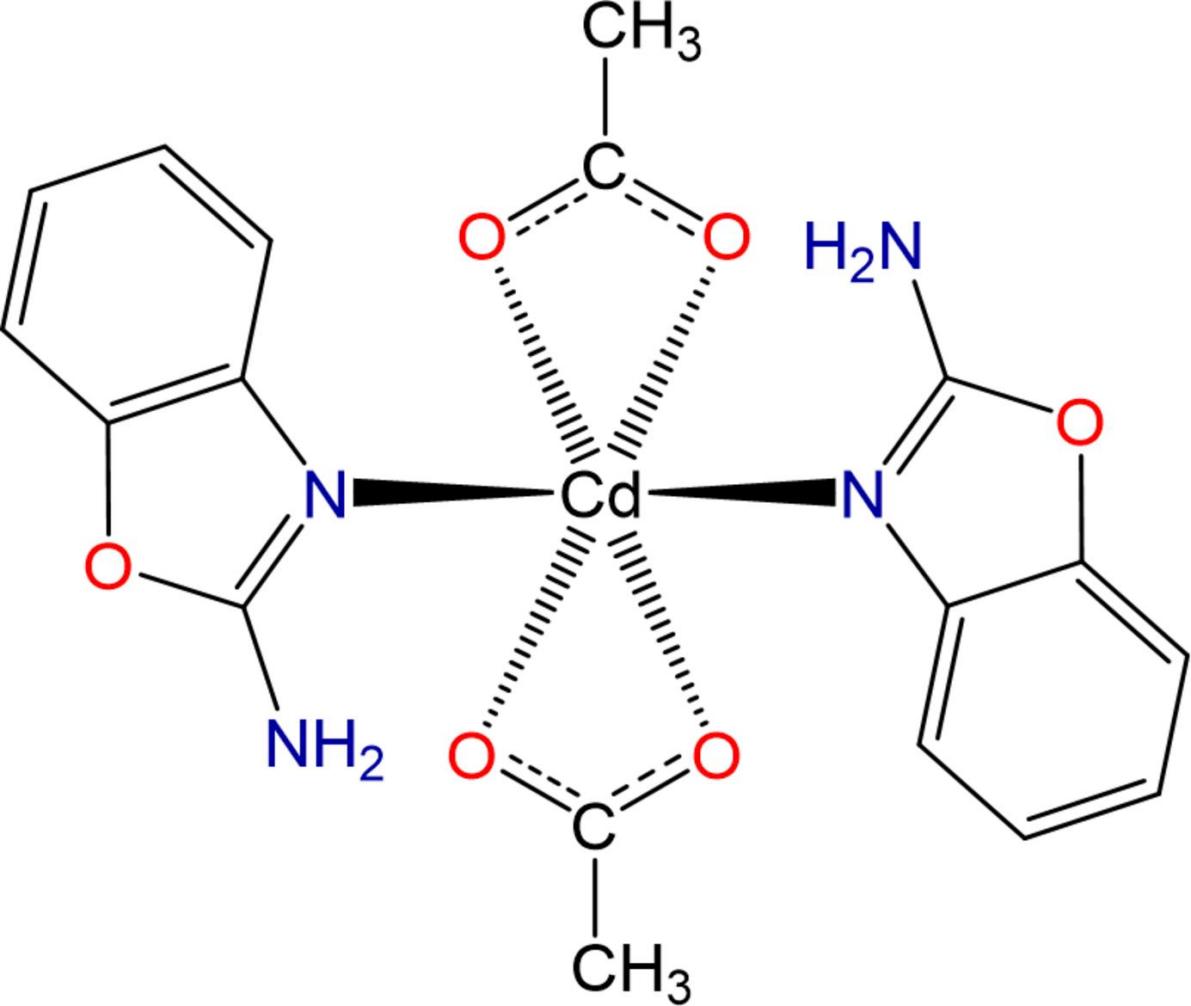


## 2. Structural commentary

**Figure 1:**
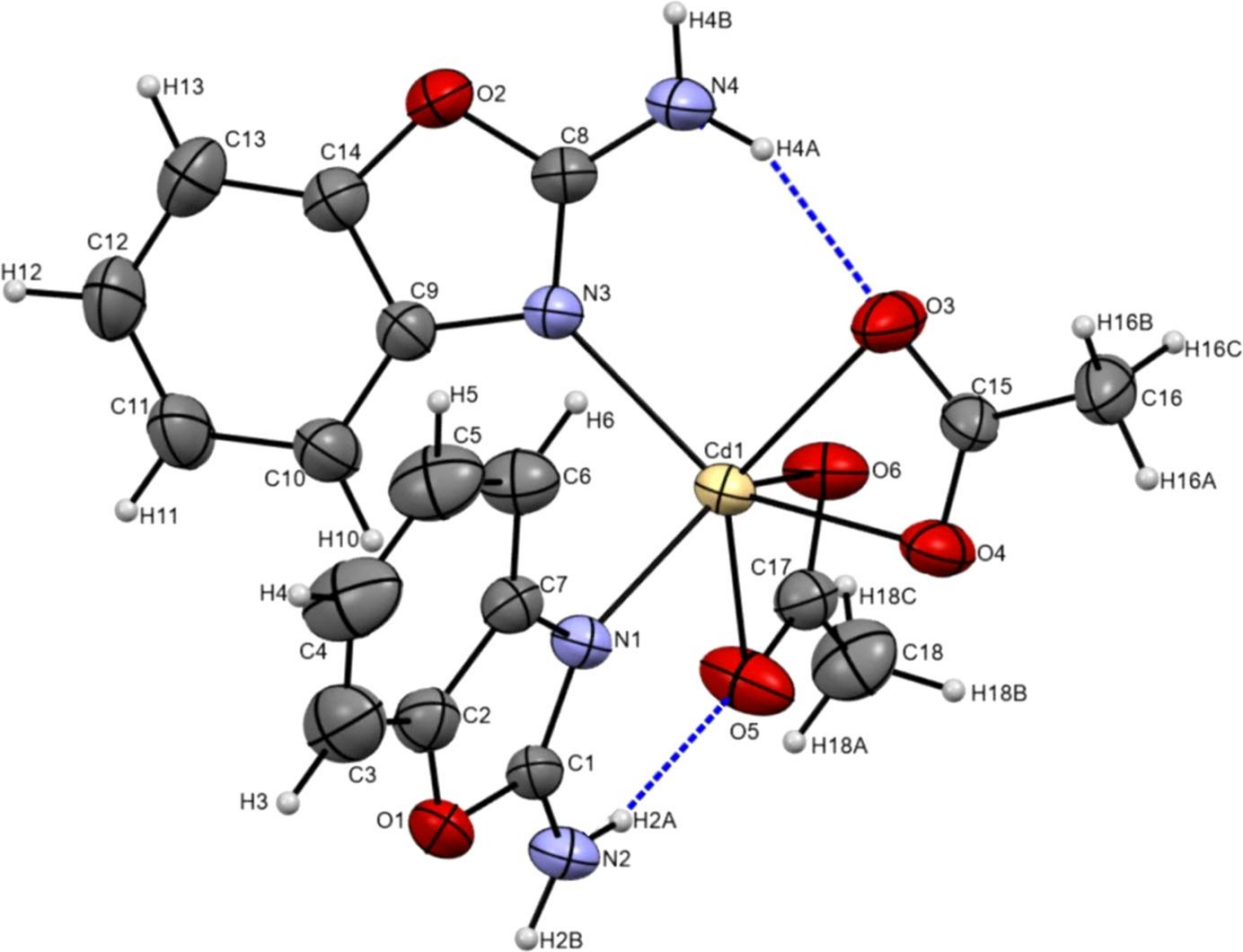
The mol­ecular structure of the title complex with the atom-numbering scheme. Intra­molecular hydrogen bonds are indicated by dashed lines. Displacement ellipsoids are plotted at the 30% probability level.

The structure of [Cd^2+^(CH_3_COO^−^)_2_(**2AB**)_2_] is shown in Fig. 1 ▸. The metal complex was obtained using the Cd(CH_3_COO)_2_ salt for the synthesis. The Cd^II^ ion coordinates two **2AB** mol­ecules through the oxazole N atom in a monodentate fashion. Furthermore, in order to compensate the positive charge of the central atom, two acetate ligands are coordinated in a bidentate manner through the O atoms. Despite the large ionic radius of the Cd atom, the coordination number of the central atom is six, in contrast to, for example, coordination numbers of four or eight in some mixed-ligand cadmium complexes (Kudiyarova *et al.*, 2021 ▸; Ibragimov *et al.*, 2017*a* ▸). The two **2AB** ligands and the two acetate ions are coordinated to the Cd centre in a *cis* arrangement. The bond lengths of the Cd ion are in the range 2.269 (2)–2.400 (2) Å, while the bond angles vary from 53.35 (8) to 139.71 (8)°. Such a large difference in the valence distances and angles leads to a significant distortion of the octahedral coordination environment, caused by the acetate ligands acting as bidentate, with chelating angles of O3—Cd1—O4 = 53.57 (8)° and O5—Cd1—O6 = 53.35 (8)° of the cadmium polyhedron. The geo­metric parameters of the arene ring are similar to standard values and to those in other structures (Ibragimov *et al.*, 2017*b* ▸; Ruzmetov *et al.*, 2022 ▸). In the **2AB** mol­ecules, all the atoms are located on a plane, with the greatest r.m.s. deviations from the main planes seen for the amino atoms N2 (0.017 Å) and N4 (0.026 Å). The dihedral angle between the mean planes of the **2AB** mol­ecules around the cadmium polyhedron is 65.59°. The positions of the ligands allow the formation of two relatively strong intra­molecular hydrogen bonds in the complex mol­ecule: in particular, the amino groups N2H_2_ and N4H_2_ form hydrogen bonds with the nearest O atoms, O5 and O3, of the coordinated acetates, with distances of 2.762 (4) and 2.790 (4) Å, respectively (Table 1 ▸). These hydrogen bonds enclose six-membered rings with *S*(6) graph-set notations (Etter, 1990 ▸).

## 3. Supra­molecular features

**Figure 2:**
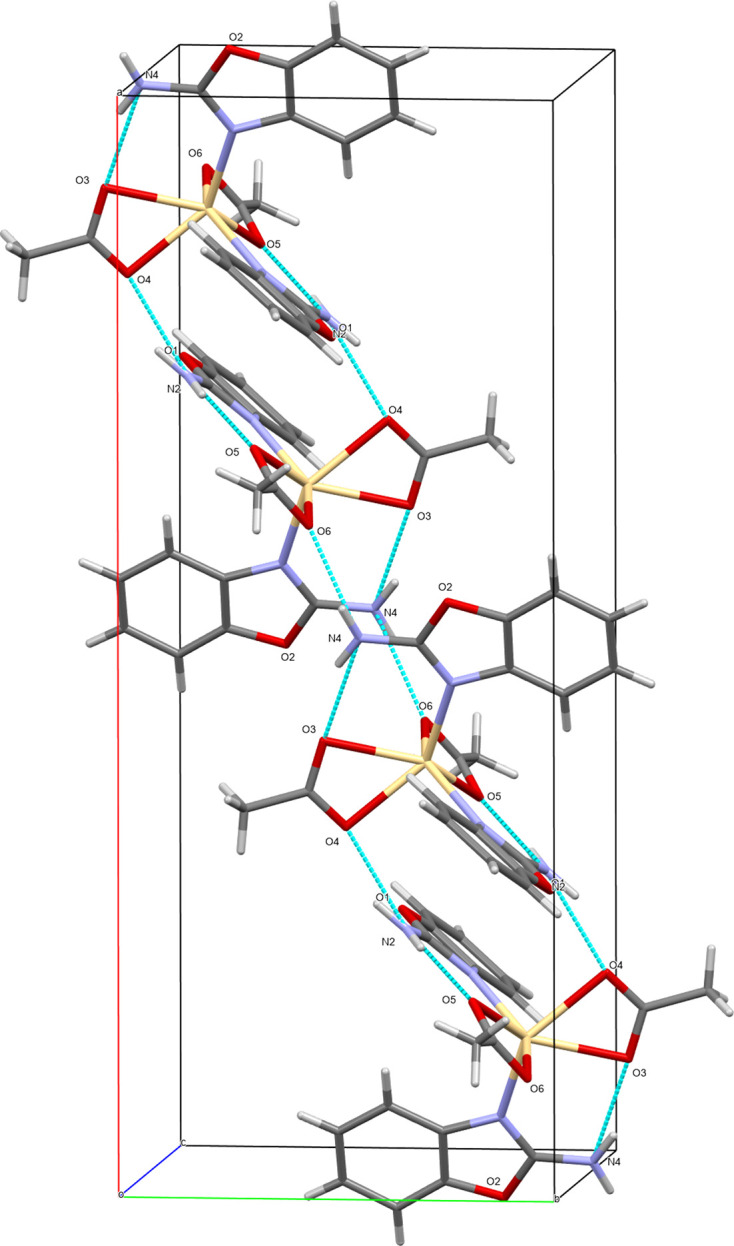
The formation of columns by hydrogen bonds in the crystal structure of the title complex. Generic atom labels without symmetry codes have been used.

**Figure 3:**
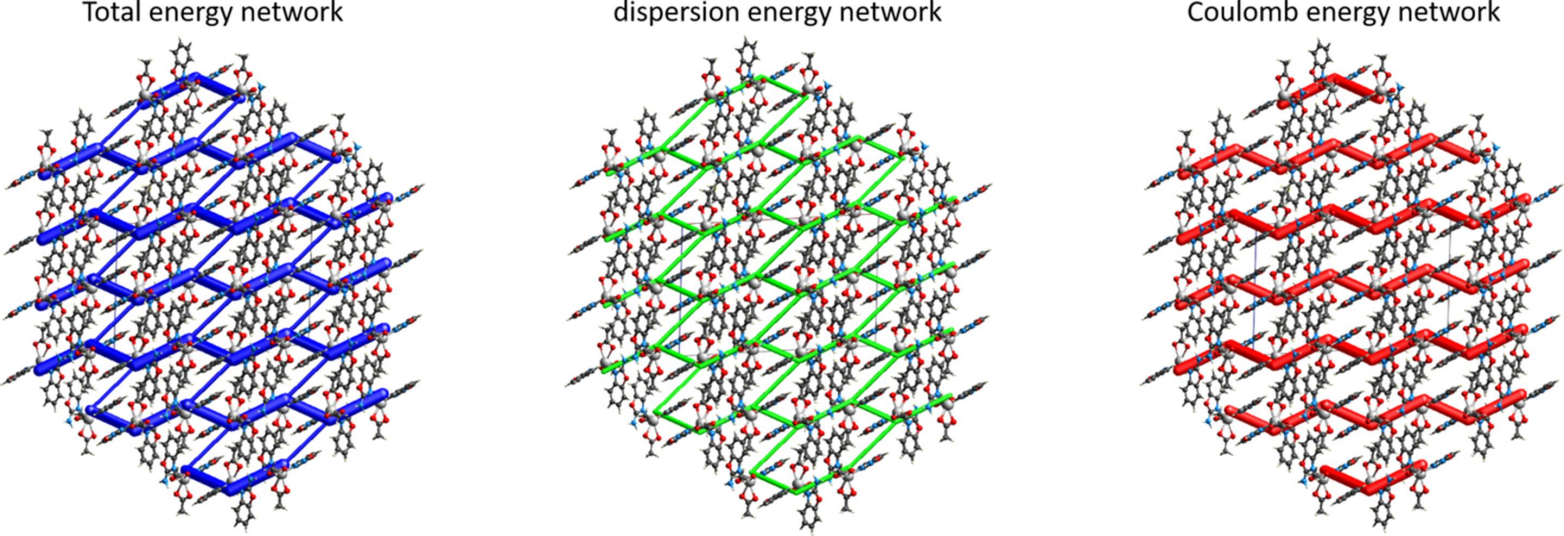
Inter­action energy calculations within the mol­ecules was performed using the HF method (HF/3-21G) in the *CrystalExplorer17* program. The thickness of the tube represents the value of the energy. The distribution of the inter­actions according to type shows strong inter­actions along the crystallographic *a* direction (the largest values are represented here). The total energy framework (in blue) and its two main components, dispersion (in green) and Coulombic energy (in red), are shown for a cluster around a reference molecule. also exhibit stronger interactions along the crystallographic *a* direction.

There are two proton-donor hydrogen-bonding groups in the complex mol­ecule, *i.e.* N2—H2 and N4—H4. Both of these groups realize their hydrogen-bonding capabilities by forming intra­molecular N2—H2*A*⋯O5 and N4—H4*A*⋯O3 (first two hydrogen bonds in Table 1 ▸), and two inter­molecular N2—H2*B*⋯O4^i^ and N4—H4*B*⋯O6^ii^ hydrogen bonds (the remaining two hydrogen bonds in Table 1 ▸). These inter­molecular hydrogen bonds between the N atoms of the amino groups and the O atoms of the acetate carboxyl­ate groups associate complex mol­ecules into columns running in the [1 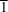 0] and [110] directions (Fig. 2 ▸). The interaction energies of the hydrogen-bond system were calculated within the mol­ecules using the HF method (HF/3-21G) in the *CrystalExplorer17* program (Fig. 3 ▸). The result shows the total energy (*E*_tot_), which is the sum of the Coulombic (*E*_ele_), polar (*E*_pol_), dispersion (*E*_dis_) and repulsive (*E*_rep_) contributions. The four energy components were scaled in the total energy (*E*_tot_ = 1.019*E*_ele_ + 0651*E*_pol_ + 0901*E*_dis_ + 0.811*E*_rep_). The inter­action energies were investigated for a 3.8 Å cluster around the reference mol­ecule. The calculation reveals two stronger inter­actions within the neighbouring mol­ecules. The strongest inter­action total energy (*E*_tot_) is −113.4 kJ mol^−1^ (∼ −27 kcal mol^−1^), with the highest polar (−32.5 kJ mol^−1^), dispersion (−51.3 kJ mol^−1^) and repulsive (68.1 kJ mol^−1^) energies (green–yellow). The second inter­action among neighbouring mol­ecules is similar to the first, with *E*_tot_ = −97.2 kJ mol^−1^. The main attractive inter­actions (Coulombic, dispersion and the sum total energy) show a stronger bonding effect along the crystallographic *a* direction (Fig. 3 ▸).

## 4. Hirshfeld surface analysis

**Figure 4:**
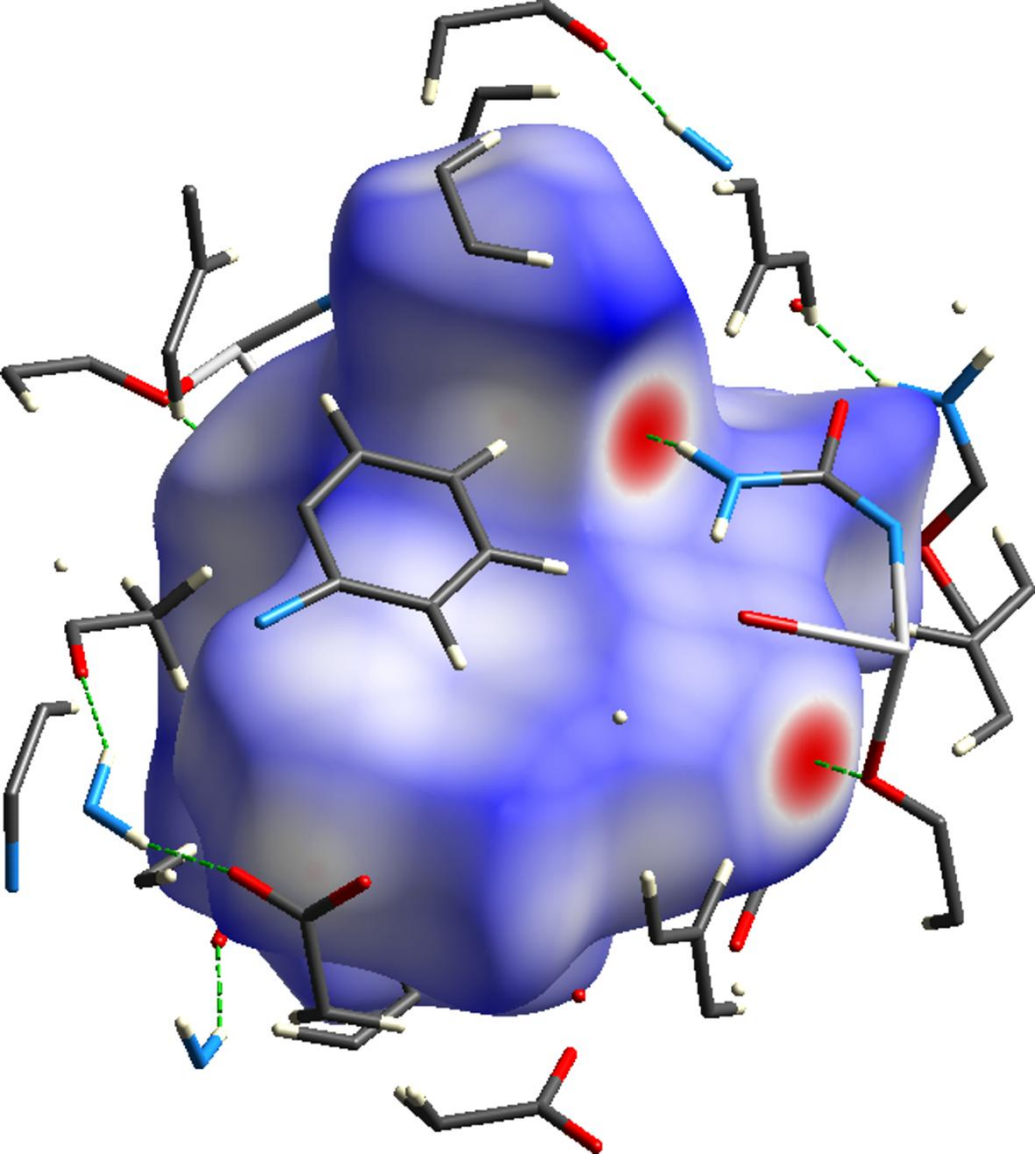
View of the three-dimensional Hirshfeld surface of the complex plotted over *d*_norm_ in the range from −0.6027 to 1.5939 a.u.

**Figure 5:**
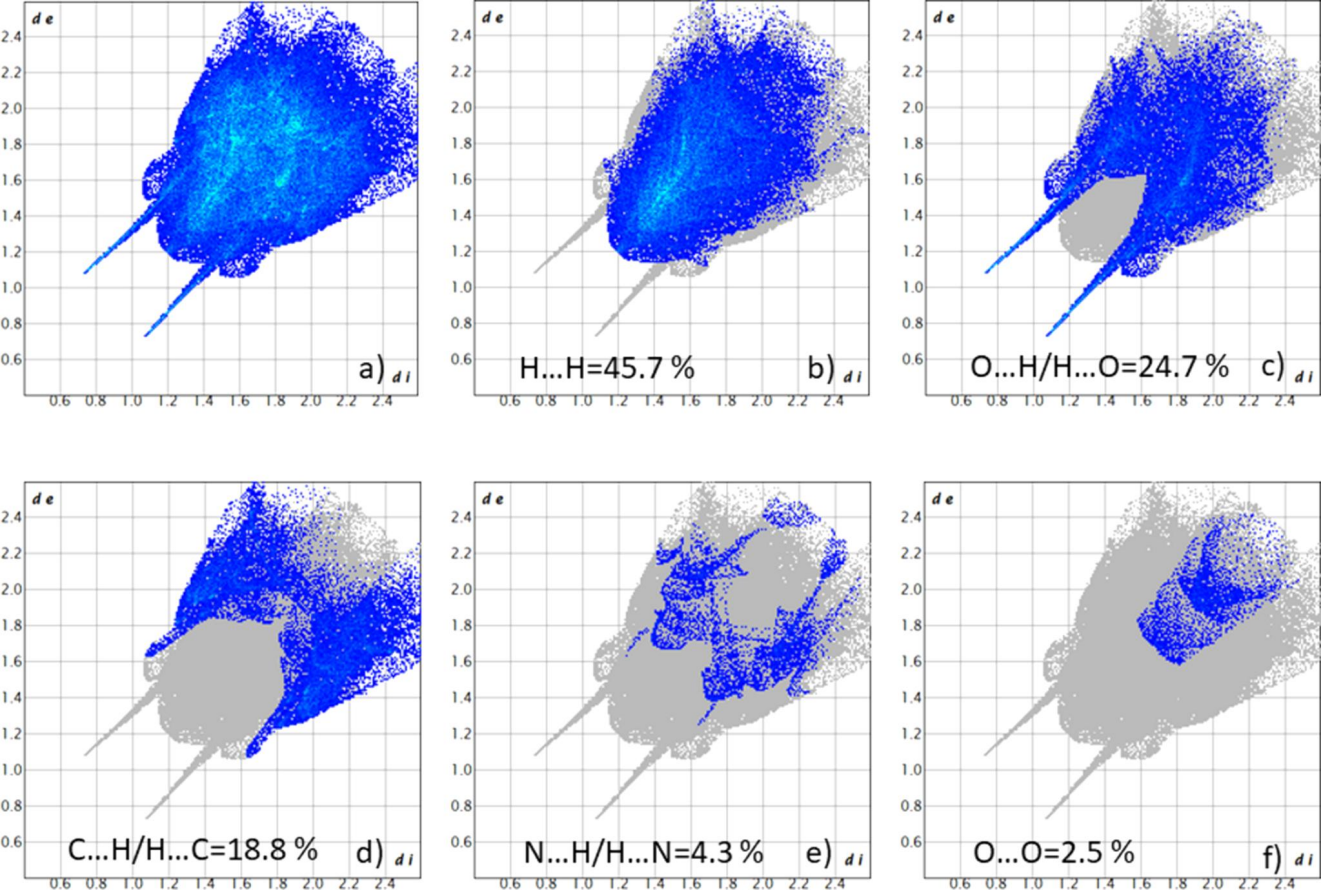
The full 2D fingerprint plots for the title complex, showing all inter­actions and delineated into separate inter­actions. The *d*_i_ and *d*_e_ values are the closest inter­nal and external distances (Å) from given points on the Hirshfeld surface contacts.

To further investigate the inter­molecular inter­actions present in the title compound, a Hirshfeld surface analysis was performed, and the two-dimensional (2D) fingerprint plots were generated with *CrystalExplorer17* (Spackman *et al.*, 2021 ▸). Fig. 4 ▸ shows the three-dimensional (3D) Hirshfeld surface of the complex with *d*_norm_ (normalized contact distance) plotted over the range from −0.6027 to 1.5939 a.u. The hydrogen-bond inter­actions given in Table 1 ▸ play a key role in the mol­ecular packing of the complex. The overall 2D fingerprint plot and those delineated into H⋯H, O⋯H/H⋯O, C⋯H/H⋯C, N⋯H/H⋯N and O⋯O inter­actions, are shown in Fig. 5 ▸. The percentage contributions to the Hirshfeld surfaces from the various inter­atomic contacts are as follows: H⋯H 45.7%, O⋯H/H⋯O 24.7%, C⋯H/H⋯C 18.8%, N⋯H/H⋯N 4.3% and O⋯O 2.5%. Other minor contributions to the Hirshfeld surface are: C⋯C 2.4% and O⋯C/C⋯O 1.6%.

## 5. Database survey

A search of the Cambridge Structural Database (CSD, Version 5.43, update of March 2022; Groom *et al.*, 2016 ▸) for free **2AB** and its metal complexes gave no hits. Cadmium(II) acetate complexes of the general formula [Cd(OAc)_2_ *L*_2_], where cadmium is hexa­coordinated, the acetate ligand is attached to cadmium in a bidentate manner and *L* is a monodentate ligand with a ligator N atom, have been reported in the CSD with refcodes ODONEC (Ma *et al.*, 2012 ▸), ODONEC01 (Yan *et al.*, 2014 ▸), PIBMIX (Zhao *et al.*, 2007 ▸), TICDOY (Chotalia *et al.*, 1996 ▸), TICMID (Hei *et al.*, 2013 ▸) and UGOPOX (Liu *et al.*, 2015 ▸).

## 6. Synthesis and crystallization

Cd(CH_3_COO)_2_·2H_2_O (0.266 g, 1 mmol) and **2AB** (0.268 g, 2 mmol) were dissolved separately in ethanol (5 ml), mixed together and stirred for 1.5 h. The obtained colourless solution was filtered and left for crystallization. Single crystals of the title complex suitable for X-ray analysis were obtained by slow evaporation of the solution over a period of 10 d.

## 7. Refinement

Crystal data, data collection and structure refinement details are summarized in Table 2 ▸. The H atoms of the acetate methyl groups were placed in calculated positions and refined in the riding-model approximation, with *U*_iso_(H) = 1.5*U*_eq_(C) and C—H = 0.96 Å. The remaining H atoms were located experimentally and refined freely.

## Supplementary Material

Crystal structure: contains datablock(s) I, global. DOI: 10.1107/S2056989023007399/jy2035sup1.cif

Structure factors: contains datablock(s) I. DOI: 10.1107/S2056989023007399/jy2035Isup2.hkl

CCDC reference: 2290113

Additional supporting information:  crystallographic information; 3D view; checkCIF report

## Figures and Tables

**Table 1:** Hydrogen-bond geometry (Å, °)

*D*—H⋯*A*	*D*—H	H⋯*A*	*D*⋯*A*	*D*—H⋯*A*
N2—H2*A*⋯O5	0.86	1.95	2.762 (4)	157
N2—H2*B*⋯O4^i^	0.86	2.04	2.811 (3)	149
N4—H4*A*⋯O3	0.86	1.99	2.790 (4)	155
N4—H4*B*⋯O6^ii^	0.86	2.04	2.803 (3)	148

Symmetry codes: (i) 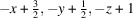 ; (ii) 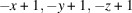 .

**Table 2:** Experimental details

Crystal data	
Chemical formula	[Cd(C_2_H_3_O_2_)_2_(C_7_H_6_N_2_O)_2_]
*M*_r_	498.76
Crystal system, space group	Monoclinic, *C*2/*c*
Temperature (K)	293
*a*, *b*, *c* (Å)	25.0497 (3), 9.8428 (1), 16.7577 (2)
β (°)	94.534 (1)
*V* (Å^3^)	4118.83 (8)
*Z*	8
Radiation type	Cu *K*α
μ (mm^−1^)	8.87
Crystal size (mm)	0.17 × 0.14 × 0.12

Data collection	
Diffractometer	Rigaku XtaLAB Synergy diffrac­tometer with a HyPix3000 detector
Absorption correction	Multi-scan (*CrysAlis PRO*; Rigaku OD, 2020 ▸)
*T*_min_, *T*_max_	0.793, 1.000
No. of measured, independent and observed [*I* > 2σ(*I*)] reflections	11316, 3971, 3434
*R*_int_	0.030
(sin θ/λ)_max_ (Å^−1^)	0.614

Refinement	
*R*[*F* ^2^ > 2σ(*F* ^2^)], *wR*(*F* ^2^), *S*	0.030, 0.078, 1.05
No. of reflections	3971
No. of parameters	265
H-atom treatment	H-atom parameters constrained
Δρ_max_, Δρ_min_ (e Å^−3^)	0.26, −0.59

Computer programs: *CrysAlis PRO* (Rigaku OD, 2020 ▸), *SHELXT* (Sheldrick, 2015*a* ▸), *SHELXL2016* (Sheldrick, 2015*b* ▸) and *OLEX2* (Dolomanov *et al.*, 2009 ▸).

## supplementary crystallographic information

### Crystal data

**Table:**

[Cd(C_2_H_3_O_2_)_2_(C_7_H_6_N_2_O)_2_]	*F*(000) = 2000
*M**_r_* = 498.76	*D*_x_ = 1.609 Mg m^−^^3^
Monoclinic, *C*2/*c*	Cu *K*α radiation, λ = 1.54184 Å
*a* = 25.0497 (3) Å	Cell parameters from 6254 reflections
*b* = 9.8428 (1) Å	θ = 3.5–70.8°
*c* = 16.7577 (2) Å	µ = 8.87 mm^−^^1^
β = 94.534 (1)°	*T* = 293 K
*V* = 4118.83 (8) Å^3^	Block, colourless
*Z* = 8	0.17 × 0.14 × 0.12 mm

### Data collection

**Table:**

Rigaku XtaLAB Synergy diffractometer with a HyPix3000 detector	3971 independent reflections
Radiation source: micro-focus sealed X-ray tube, PhotonJet (Cu) X-ray Source	3434 reflections with *I* > 2σ(*I*)
Mirror monochromator	*R*_int_ = 0.030
Detector resolution: 10.0000 pixels mm^-1^	θ_max_ = 71.2°, θ_min_ = 3.5°
ω scans	*h* = −30→30
Absorption correction: multi-scan (CrysAlis PRO; Rigaku OD, 2020)	*k* = −12→11
*T*_min_ = 0.793, *T*_max_ = 1.000	*l* = −20→20
11316 measured reflections	

### Refinement

**Table:**

Refinement on *F*^2^	Hydrogen site location: inferred from neighbouring sites
Least-squares matrix: full	H-atom parameters constrained
*R*[*F*^2^ > 2σ(*F*^2^)] = 0.030	*w* = 1/[σ^2^(*F*_o_^2^) + (0.0384*P*)^2^ + 0.4924*P*] where *P* = (*F*_o_^2^ + 2*F*_c_^2^)/3
*wR*(*F*^2^) = 0.078	(Δ/σ)_max_ = 0.002
*S* = 1.05	Δρ_max_ = 0.26 e Å^−^^3^
3971 reflections	Δρ_min_ = −0.59 e Å^−^^3^
265 parameters	Extinction correction: SHELXL2016 (Sheldrick, 2015b), Fc^*^=kFc[1+0.001xFc^2^λ^3^/sin(2θ)]^-1/4^
0 restraints	Extinction coefficient: 0.000082 (13)
Primary atom site location: structure-invariant direct methods	

### Special details

**Table:**

Geometry. All esds (except the esd in the dihedral angle between two l.s. planes) are estimated using the full covariance matrix. The cell esds are taken into account individually in the estimation of esds in distances, angles and torsion angles; correlations between esds in cell parameters are only used when they are defined by crystal symmetry. An approximate (isotropic) treatment of cell esds is used for estimating esds involving l.s. planes.

### Fractional atomic coordinates and isotropic or equivalent isotropic displacement parameters (Å^2^)

**Table:**

	*x*	*y*	*z*	*U*_iso_*/*U*_eq_	
Cd1	0.62410 (2)	0.36216 (2)	0.51980 (2)	0.05241 (10)	
O1	0.73371 (8)	0.0598 (2)	0.64662 (14)	0.0683 (6)	
O2	0.47350 (9)	0.2945 (3)	0.63959 (16)	0.0769 (7)	
N1	0.67802 (9)	0.2252 (2)	0.60113 (14)	0.0537 (6)	
O6	0.59362 (9)	0.3834 (3)	0.38471 (16)	0.0791 (7)	
O4	0.68374 (9)	0.5447 (2)	0.52681 (17)	0.0789 (7)	
N3	0.54967 (9)	0.2942 (3)	0.57764 (16)	0.0572 (6)	
N2	0.72228 (10)	0.0877 (3)	0.51189 (17)	0.0642 (7)	
H2A	0.707645	0.126500	0.469675	0.077*	
H2B	0.744585	0.022233	0.507514	0.077*	
O3	0.60454 (10)	0.5914 (3)	0.5565 (2)	0.0996 (10)	
O5	0.66139 (11)	0.2548 (3)	0.40824 (15)	0.0948 (9)	
C15	0.65104 (13)	0.6280 (3)	0.5472 (2)	0.0578 (7)	
C1	0.71057 (11)	0.1285 (3)	0.5831 (2)	0.0528 (7)	
N4	0.50825 (10)	0.5006 (3)	0.6106 (2)	0.0821 (9)	
H4A	0.532476	0.550805	0.591987	0.099*	
H4B	0.481505	0.537324	0.631454	0.099*	
C17	0.62821 (12)	0.3087 (3)	0.36070 (19)	0.0600 (7)	
C7	0.67858 (11)	0.2219 (3)	0.68508 (18)	0.0574 (7)	
C9	0.53519 (12)	0.1592 (3)	0.5921 (2)	0.0613 (8)	
C2	0.71305 (13)	0.1203 (4)	0.7127 (2)	0.0678 (9)	
C8	0.51215 (13)	0.3665 (3)	0.6070 (2)	0.0636 (8)	
C14	0.48834 (14)	0.1591 (4)	0.6302 (2)	0.0718 (9)	
C16	0.66689 (16)	0.7730 (3)	0.5600 (2)	0.0842 (11)	
H16A	0.704972	0.781517	0.558471	0.126*	
H16B	0.656663	0.802761	0.611189	0.126*	
H16C	0.649232	0.828000	0.518610	0.126*	
C6	0.65278 (14)	0.3000 (4)	0.7383 (2)	0.0777 (10)	
H6	0.629353	0.369251	0.721214	0.093*	
C10	0.55866 (14)	0.0371 (4)	0.5755 (3)	0.0810 (11)	
H10	0.590396	0.033844	0.550275	0.097*	
C11	0.53352 (16)	−0.0804 (4)	0.5975 (3)	0.0931 (13)	
H11	0.548254	−0.164167	0.585847	0.112*	
C13	0.46251 (17)	0.0455 (5)	0.6528 (3)	0.0977 (13)	
H13	0.430576	0.049423	0.677524	0.117*	
C18	0.63098 (17)	0.2827 (5)	0.2729 (2)	0.0957 (13)	
H18A	0.647214	0.195777	0.265397	0.144*	
H18B	0.652023	0.352341	0.250357	0.144*	
H18C	0.595468	0.283398	0.246754	0.144*	
C3	0.72412 (17)	0.0889 (6)	0.7912 (3)	0.1035 (15)	
H3	0.747541	0.019434	0.807937	0.124*	
C5	0.66360 (17)	0.2699 (6)	0.8190 (2)	0.1001 (14)	
H5	0.646981	0.320491	0.856801	0.120*	
C12	0.48703 (18)	−0.0764 (5)	0.6365 (3)	0.1025 (15)	
H12	0.471821	−0.157333	0.652136	0.123*	
C4	0.6980 (2)	0.1678 (6)	0.8445 (3)	0.1133 (18)	
H4	0.704006	0.151002	0.899045	0.136*	

### Atomic displacement parameters (Å^2^)

**Table:**

	*U*^11^	*U*^22^	*U*^33^	*U*^12^	*U*^13^	*U*^23^
Cd1	0.04847 (14)	0.04693 (14)	0.06296 (15)	0.00942 (8)	0.01144 (9)	−0.00134 (9)
O1	0.0585 (12)	0.0626 (14)	0.0837 (16)	0.0146 (10)	0.0057 (11)	0.0158 (12)
O2	0.0578 (13)	0.0723 (16)	0.1046 (19)	0.0008 (11)	0.0321 (12)	0.0026 (14)
N1	0.0502 (12)	0.0513 (14)	0.0604 (14)	0.0101 (11)	0.0094 (10)	0.0017 (12)
O6	0.0599 (13)	0.0937 (18)	0.0844 (17)	0.0208 (12)	0.0096 (12)	−0.0112 (14)
O4	0.0608 (13)	0.0584 (13)	0.119 (2)	0.0111 (11)	0.0163 (12)	−0.0188 (14)
N3	0.0455 (12)	0.0487 (13)	0.0788 (17)	0.0061 (11)	0.0131 (11)	0.0024 (13)
N2	0.0586 (14)	0.0593 (15)	0.0760 (18)	0.0178 (13)	0.0130 (12)	−0.0037 (14)
O3	0.0752 (16)	0.0610 (14)	0.170 (3)	−0.0079 (13)	0.0582 (17)	−0.0258 (17)
O5	0.1068 (19)	0.105 (2)	0.0710 (16)	0.0516 (17)	−0.0018 (14)	−0.0074 (15)
C15	0.0681 (19)	0.0468 (16)	0.0608 (18)	0.0067 (14)	0.0200 (15)	0.0019 (13)
C1	0.0418 (14)	0.0471 (15)	0.0697 (19)	0.0016 (12)	0.0069 (13)	0.0049 (14)
N4	0.0599 (16)	0.0578 (16)	0.133 (3)	0.0145 (13)	0.0356 (16)	−0.0006 (18)
C17	0.0547 (16)	0.0640 (19)	0.0621 (18)	0.0020 (15)	0.0092 (14)	0.0002 (16)
C7	0.0483 (15)	0.0618 (18)	0.0629 (18)	−0.0014 (14)	0.0087 (13)	0.0031 (16)
C9	0.0523 (17)	0.0525 (18)	0.079 (2)	0.0005 (13)	0.0049 (15)	0.0044 (16)
C2	0.0567 (18)	0.077 (2)	0.070 (2)	0.0027 (16)	0.0054 (15)	0.0133 (18)
C8	0.0509 (17)	0.062 (2)	0.080 (2)	0.0074 (14)	0.0151 (15)	0.0054 (16)
C14	0.0602 (19)	0.066 (2)	0.090 (3)	0.0000 (16)	0.0145 (17)	0.0017 (19)
C16	0.095 (3)	0.052 (2)	0.111 (3)	−0.0050 (18)	0.042 (2)	−0.005 (2)
C6	0.071 (2)	0.091 (3)	0.072 (2)	0.009 (2)	0.0092 (17)	−0.007 (2)
C10	0.062 (2)	0.058 (2)	0.122 (3)	0.0036 (16)	0.0091 (19)	−0.001 (2)
C11	0.079 (3)	0.055 (2)	0.144 (4)	−0.0059 (19)	0.005 (3)	0.004 (2)
C13	0.077 (2)	0.092 (3)	0.127 (4)	−0.021 (2)	0.027 (2)	0.011 (3)
C18	0.105 (3)	0.122 (4)	0.061 (2)	−0.002 (3)	0.016 (2)	−0.006 (2)
C3	0.089 (3)	0.132 (4)	0.088 (3)	0.018 (3)	0.001 (2)	0.037 (3)
C5	0.092 (3)	0.143 (4)	0.067 (2)	0.007 (3)	0.013 (2)	−0.010 (3)
C12	0.091 (3)	0.068 (3)	0.149 (5)	−0.020 (2)	0.016 (3)	0.016 (3)
C4	0.094 (3)	0.185 (6)	0.062 (2)	0.012 (3)	0.008 (2)	0.017 (3)

### Geometric parameters (Å, º)

**Table:**

Cd1—N1	2.282 (2)	C17—C18	1.501 (5)
Cd1—O6	2.341 (3)	C7—C2	1.377 (4)
Cd1—O4	2.333 (2)	C7—C6	1.377 (4)
Cd1—N3	2.269 (2)	C9—C14	1.380 (5)
Cd1—O3	2.399 (3)	C9—C10	1.376 (5)
Cd1—O5	2.400 (2)	C2—C3	1.358 (5)
Cd1—C15	2.732 (3)	C14—C13	1.360 (5)
Cd1—C17	2.727 (3)	C16—H16A	0.9600
O1—C1	1.353 (4)	C16—H16B	0.9600
O1—C2	1.392 (4)	C16—H16C	0.9600
O2—C8	1.349 (4)	C6—H6	0.9300
O2—C14	1.396 (4)	C6—C5	1.389 (5)
N1—C1	1.304 (4)	C10—H10	0.9300
N1—C7	1.406 (4)	C10—C11	1.381 (5)
O6—C17	1.228 (4)	C11—H11	0.9300
O4—C15	1.227 (4)	C11—C12	1.380 (6)
N3—C9	1.404 (4)	C13—H13	0.9300
N3—C8	1.306 (4)	C13—C12	1.385 (6)
N2—H2A	0.8600	C18—H18A	0.9600
N2—H2B	0.8600	C18—H18B	0.9600
N2—C1	1.314 (4)	C18—H18C	0.9600
O3—C15	1.241 (4)	C3—H3	0.9300
O5—C17	1.226 (4)	C3—C4	1.386 (7)
C15—C16	1.492 (4)	C5—H5	0.9300
N4—H4A	0.8600	C5—C4	1.369 (7)
N4—H4B	0.8600	C12—H12	0.9300
N4—C8	1.325 (4)	C4—H4	0.9300
			
N1—Cd1—O6	139.71 (8)	O6—C17—C18	120.8 (3)
N1—Cd1—O4	94.49 (8)	O5—C17—Cd1	61.59 (18)
N1—Cd1—O3	121.70 (11)	O5—C17—O6	120.4 (3)
N1—Cd1—O5	87.52 (8)	O5—C17—C18	118.8 (3)
N1—Cd1—C15	109.79 (9)	C18—C17—Cd1	178.6 (3)
N1—Cd1—C17	113.80 (9)	C2—C7—N1	108.0 (3)
O6—Cd1—O3	96.12 (10)	C6—C7—N1	131.9 (3)
O6—Cd1—O5	53.35 (8)	C6—C7—C2	120.1 (3)
O6—Cd1—C15	97.73 (9)	C14—C9—N3	108.8 (3)
O6—Cd1—C17	26.66 (8)	C10—C9—N3	132.2 (3)
O4—Cd1—O6	97.96 (10)	C10—C9—C14	119.1 (3)
O4—Cd1—O3	53.57 (8)	C7—C2—O1	107.8 (3)
O4—Cd1—O5	95.22 (10)	C3—C2—O1	127.8 (3)
O4—Cd1—C15	26.57 (8)	C3—C2—C7	124.4 (4)
O4—Cd1—C17	97.13 (10)	N3—C8—O2	115.3 (3)
N3—Cd1—N1	92.19 (9)	N3—C8—N4	128.1 (3)
N3—Cd1—O6	102.90 (9)	N4—C8—O2	116.5 (3)
N3—Cd1—O4	138.59 (9)	C9—C14—O2	107.2 (3)
N3—Cd1—O3	88.65 (9)	C13—C14—O2	128.1 (4)
N3—Cd1—O5	125.90 (11)	C13—C14—C9	124.8 (4)
N3—Cd1—C15	114.27 (9)	C15—C16—H16A	109.5
N3—Cd1—C17	117.16 (9)	C15—C16—H16B	109.5
O3—Cd1—O5	135.54 (11)	C15—C16—H16C	109.5
O3—Cd1—C15	27.00 (9)	H16A—C16—H16B	109.5
O3—Cd1—C17	117.15 (11)	H16A—C16—H16C	109.5
O5—Cd1—C15	116.52 (11)	H16B—C16—H16C	109.5
O5—Cd1—C17	26.69 (8)	C7—C6—H6	121.8
C17—Cd1—C15	108.73 (10)	C7—C6—C5	116.5 (4)
C1—O1—C2	104.4 (2)	C5—C6—H6	121.8
C8—O2—C14	104.5 (2)	C9—C10—H10	121.1
C1—N1—Cd1	130.1 (2)	C9—C10—C11	117.8 (4)
C1—N1—C7	105.0 (2)	C11—C10—H10	121.1
C7—N1—Cd1	124.74 (18)	C10—C11—H11	119.3
C17—O6—Cd1	94.5 (2)	C12—C11—C10	121.4 (4)
C15—O4—Cd1	95.2 (2)	C12—C11—H11	119.3
C9—N3—Cd1	125.8 (2)	C14—C13—H13	122.3
C8—N3—Cd1	129.8 (2)	C14—C13—C12	115.4 (4)
C8—N3—C9	104.3 (3)	C12—C13—H13	122.3
H2A—N2—H2B	120.0	C17—C18—H18A	109.5
C1—N2—H2A	120.0	C17—C18—H18B	109.5
C1—N2—H2B	120.0	C17—C18—H18C	109.5
C15—O3—Cd1	91.61 (19)	H18A—C18—H18B	109.5
C17—O5—Cd1	91.7 (2)	H18A—C18—H18C	109.5
O4—C15—Cd1	58.28 (16)	H18B—C18—H18C	109.5
O4—C15—O3	119.7 (3)	C2—C3—H3	122.4
O4—C15—C16	120.2 (3)	C2—C3—C4	115.2 (4)
O3—C15—Cd1	61.39 (17)	C4—C3—H3	122.4
O3—C15—C16	120.1 (3)	C6—C5—H5	119.0
C16—C15—Cd1	178.1 (3)	C4—C5—C6	122.0 (4)
N1—C1—O1	114.8 (3)	C4—C5—H5	119.0
N1—C1—N2	128.5 (3)	C11—C12—C13	121.5 (4)
N2—C1—O1	116.7 (3)	C11—C12—H12	119.2
H4A—N4—H4B	120.0	C13—C12—H12	119.2
C8—N4—H4A	120.0	C3—C4—H4	119.1
C8—N4—H4B	120.0	C5—C4—C3	121.8 (4)
O6—C17—Cd1	58.82 (18)	C5—C4—H4	119.1
			
Cd1—N1—C1—O1	−175.14 (18)	C1—N1—C7—C6	179.2 (3)
Cd1—N1—C1—N2	2.7 (5)	C7—N1—C1—O1	−0.4 (3)
Cd1—N1—C7—C2	175.5 (2)	C7—N1—C1—N2	177.4 (3)
Cd1—N1—C7—C6	−5.7 (5)	C7—C2—C3—C4	−0.2 (7)
Cd1—O6—C17—O5	1.0 (4)	C7—C6—C5—C4	−0.1 (7)
Cd1—O6—C17—C18	−178.5 (3)	C9—N3—C8—O2	−0.4 (4)
Cd1—O4—C15—O3	0.7 (4)	C9—N3—C8—N4	177.7 (4)
Cd1—O4—C15—C16	−178.7 (3)	C9—C14—C13—C12	1.3 (7)
Cd1—N3—C9—C14	178.2 (2)	C9—C10—C11—C12	−1.4 (7)
Cd1—N3—C9—C10	−1.8 (6)	C2—O1—C1—N1	0.2 (3)
Cd1—N3—C8—O2	−178.2 (2)	C2—O1—C1—N2	−177.9 (3)
Cd1—N3—C8—N4	−0.2 (6)	C2—C7—C6—C5	−0.1 (5)
Cd1—O3—C15—O4	−0.6 (4)	C2—C3—C4—C5	0.0 (8)
Cd1—O3—C15—C16	178.7 (3)	C8—O2—C14—C9	−0.2 (4)
Cd1—O5—C17—O6	−1.0 (4)	C8—O2—C14—C13	−179.6 (4)
Cd1—O5—C17—C18	178.5 (3)	C8—N3—C9—C14	0.2 (4)
O1—C2—C3—C4	179.2 (4)	C8—N3—C9—C10	−179.8 (4)
O2—C14—C13—C12	−179.4 (4)	C14—O2—C8—N3	0.4 (4)
N1—C7—C2—O1	−0.3 (4)	C14—O2—C8—N4	−177.9 (4)
N1—C7—C2—C3	179.1 (4)	C14—C9—C10—C11	0.5 (6)
N1—C7—C6—C5	−178.7 (4)	C14—C13—C12—C11	−2.1 (8)
N3—C9—C14—O2	0.0 (4)	C6—C7—C2—O1	−179.2 (3)
N3—C9—C14—C13	179.5 (4)	C6—C7—C2—C3	0.2 (6)
N3—C9—C10—C11	−179.5 (4)	C6—C5—C4—C3	0.2 (8)
C1—O1—C2—C7	0.1 (3)	C10—C9—C14—O2	180.0 (3)
C1—O1—C2—C3	−179.4 (4)	C10—C9—C14—C13	−0.5 (6)
C1—N1—C7—C2	0.5 (3)	C10—C11—C12—C13	2.2 (8)

### Hydrogen-bond geometry (Å, º)

**Table:**

*D*—H···*A*	*D*—H	H···*A*	*D*···*A*	*D*—H···*A*
N2—H2*A*···O5	0.86	1.95	2.762 (4)	157
N2—H2*B*···O4^i^	0.86	2.04	2.811 (3)	149
N4—H4*A*···O3	0.86	1.99	2.790 (4)	155
N4—H4*B*···O6^ii^	0.86	2.04	2.803 (3)	148

Symmetry codes: (i) −*x*+3/2, −*y*+1/2, −*z*+1; (ii) −*x*+1, −*y*+1, −*z*+1.
